# Independent Component Analysis of Instantaneous Power-Based fMRI

**DOI:** 10.1155/2014/579652

**Published:** 2014-03-06

**Authors:** Yuan Zhong, Gang Zheng, Yijun Liu, Guangming Lu

**Affiliations:** ^1^School of Psychology, Nanjing Normal University, Nanjing 210097, China; ^2^Department of Medical Imaging, Jinling Hospital, Nanjing University School of Medicine, Nanjing 210002, China; ^3^Departments of Psychiatry and Neuroscience, McKnight Brain Institute, University of Florida, Gainesville, FL 32610, USA

## Abstract

In functional magnetic resonance imaging (fMRI) studies using spatial independent component analysis (sICA) method, a model of “latent variables” is often employed, which is based on the assumption that fMRI data are linear mixtures of statistically independent signals. However, actual fMRI signals are nonlinear and do not automatically meet with the requirement of sICA. To provide a better solution to this problem, we proposed a novel approach termed instantaneous power based fMRI (ip-fMRI) for regularization of fMRI data. Given that the instantaneous power of fMRI signals is a scalar value, it should be a linear mixture that naturally satisfies the “latent variables” model. Based on our simulated data, the curves of accuracy and resulting receiver-operating characteristic curves indicate that the proposed approach is superior to the traditional fMRI in terms of accuracy and specificity by using sICA. Experimental results from human subjects have shown that spatial components of a hand movement task-induced activation reveal a brain network more specific to motor function by ip-fMRI than that by the traditional fMRI. We conclude that ICA decomposition of ip-fMRI may be used to localize energy signal changes in the brain and may have a potential to be applied to detection of brain activity.

## 1. Introduction

Independent component analysis (ICA) is a data-driven approach that uses higher-order statistical moments to provide solutions to blind-source separation problems [1]. This technique has been shown to be capable of partitioning various physiological and physical signals in functional magnetic resonance imaging (fMRI) studies of brain activation [2–5]. There are primarily two types of ICA methods: (i) temporal ICA (tICA), which is used to detect specific changes in the time series of fMRI signals from brain regions of interest (ROIs) [6], and (ii) spatial ICA (sICA), which is often used to localize brain activity changes and has, so far, been the dominant ICA method used in fMRI applications [7]. Here, we focus only on the sICA method for fMRI data analysis.

In fMRI data, blood oxygenation level-dependent (BOLD) signals represent brain activity changes and can be expressed by fluctuations of *T*_2_* signals [8]. The BOLD signal is a complex function of neural activity, oxygen metabolism, cerebral blood volume, cerebral blood flow, and other physiological parameters. A basic assumption of sICA for fMRI data analysis is that the observed fMRI signals are a linear sum of various components separated at each voxel [9]. However, The dynamics underlying neural activity and hemodynamic physiology are believed to be nonlinear [10, 11], and they do not automatically satisfy the commonly used latent variables model (see below). Although there are always computational solutions of sICA for fMRI data, they are nonunique [12]. Hence, in order to use sICA for localization of brain activity changes, the problem of converting nonlinear signals to linear ones needs to be solved.

Although unique nonlinear ICA has been proposed in previous studies [13] and there are different approaches for regularizing ICA solutions [12, 14], so far these methods have rarely been applied to fMRI studies. For example, the methods for transforming postnonlinear mixtures in ICA to invertible linear mixtures have been established [15, 16]. In addition, convolutive postnonlinear mixtures can be treated in the same manner as certain postnonlinear ones, which can then be transformed to linear mixtures [12]. A fundamental difficulty of the nonlinear ICA problem-solving is that its solutions are nonunique if there is no suitable regularization. More generally, a basic method for solving nonlinear ICA problems is to transform nonlinear variables or measurements into linear mixtures such that nonlinear ICA problems can be reduced to traditional linear ICA problems.

In the present study, we propose using instantaneous power as a new regularization approach in ICA for transforming nonlinear fMRI signals to linear forms in order to automatically satisfy the assumption in the “latent variable” ICA model. The instantaneous power-based fMRI (ip-fMRI) approach defines the energy of fMRI signals by their inner products such that the signal energy can be represented by a variance of fMRI signals, which is regarded as integral to their instantaneous power. Based on the regulation, the instantaneous power of fMRI signals is then partitioned into independent components using conventional sICA. There are three steps in establishing our method. First, we briefly describe the theories of ICA and the instantaneous power of fMRI signals. Second, based on the resulting accuracy and receiving operator characteristic (ROC) curves, we describe how simulated fMRI data can be used to evaluate the performance of the ip-fMRI approach and compare it with the traditional fMRI results. Third, we describe how the new approach was further tested by applying it to human data for the analysis of task-induced brain activations.

## 2. Theory

### 2.1. The Latent Variable Model of ICA

In classical ICA methods, a statistical “latent variable” model [9, 17] is often used based on the assumption that observed random variables [*x*(1, *l*),…, *x*(*t*, *l*),…, *x*(*T*, *l*)] are linear mixtures of latent variables [*s*(1, *l*),…, *s*(*m*, *l*),…, *s*(*M*, *l*)] that are non-Gaussian and mutually independent [9]. Consider the following:

(1) x(1,l)=a11s(1,l)+a12s(2,l) +⋯a1ms(m,l)+⋯a1Ms(M,l)⋮x(t,l)=at1s(1,l)+at2s(2,l) +⋯atms(m,l)+⋯atMs(M,l)⋮x(T,l)=aT1s(1,l)+aT2s(2,l) +⋯aTms(m,l)+⋯aTMs(M,l).

In the matrix notation, this can be written simply as

(2) $$\begin{array}{c} X=AS, \end{array}$$

(see [18]), where *X* = [*x*(1, *l*), *x*(2, *l*),…, *x*(*T*, *l*)]^*T*^ is a vector of observed variables, *S* = [*s*(1, *l*), *s*(2, *l*),…, *s*(*M*, *l*)]^*T*^ is a vector of latent independent sources, and *A* = [*a*_*t*1_, *a*_*t*2_,…, *a*_*tM*_] is the mixing matrix.

In sICA, the row of *X* corresponds to the voxels in fMRI signals and the column denotes time series. *l* = 1,2,…, *L* is the spatial index of voxels in one volume, where *L* is the total number of voxels, *t* = 1,2,…*T* is the temporal index of fMRI time series, and *m* = 1,2,…, *M* is the component index. In addition, the total time points of fMRI signals *T* are no less than the total number of components *M* according to the problem-solving processes of blind-source separation.

### 2.2. The Energy and the Instantaneous Power of fMRI Signals

Suppose that *P*_*x*_(1, *l*), *P*_*x*_(2, *l*),…, *P*_*x*_(*t*, *l*),…, *P*_*x*_(*T*, *l*) represent the energies of observed fMRI signals *x*(1, *l*),…, *x*(*t*, *l*),…, *x*(*T*, *l*), respectively. Because *x*(*t*, *l*) represents the signal in one voxel at time *t*, the energy term *P*_*x*_(*t*, *l*) is an instantaneous value and can be expressed by

(3) Px(t,l)=at1Ps(1,l)+at2Ps(2,l)+⋯atmPs(m,l)+⋯atMPs(M,l),

where *P*_*s*_(1, *l*), *P*_*s*_(2, *l*),…, *P*_*s*_(*m*, *l*),…*P*_*s*_(*M*, *l*) are the instantaneous powers of signals *s*(1, *l*),…, *s*(*m*, *l*),…, *s*(*M*, *l*), respectively. The overall energy of observed data in each voxel can then be represented by

(4) $$\begin{array}{c} E_{x}\left(l\right)=\overset{T}{\underset{t=1}{\sum }}P_{x}\left(t,l\right). \end{array}$$

Because the mixing matrix is a normalized weight matrix [9], the energy of observed data is nearly equal to the energy of source signals (supposing that the spatially and temporally white noise are eliminated from observed data) and can be written as

(5) $$\begin{array}{c} E_{s}\left(l\right)=\overset{M}{\underset{m=1}{\sum }}P_{s}\left(m,l\right)\approx E_{x}\left(l\right). \end{array}$$

Therefore, even if source signals are nonlinear, their energy signals *P*_*s*_(1, *l*), *P*_*s*_(2, *l*),…, *P*_*s*_(*m*, *l*),…, *P*_*s*_(*M*, *l*) are additive, which satisfies the requirement of the “latent variable” model of sICA. Then, the remaining question is how to define the energy and instantaneous power of fMRI signals.

To give the definitions, the concept of electric energy can be used as an analogy. In fMRI, if the observed *T*_2_* signals are taken as the instant voltage or current fluctuation of the resistance, we can define the energy of *T*_2_* signals by the inner product of the signals, which can be written as

(6) $$\begin{array}{c} E_{T_{2}^{*}}\left(l\right)=\langle x,x\rangle =\int x^{2}\left(t,l\right)dt. \end{array}$$

Because the energy of fMRI signals is associated with the variations of *T*_2_* signals [8], the temporal variance of *T*_2_* signals can be used to define the overall energy of fMRI signals by

(7) $$\begin{array}{c} E_{\text{BOLD}}=\int {\left[x\left(t,l\right)-\overset{-}{x}\left(l\right)\right]}^{2}dt, \end{array}$$

where *x*(*t*, *l*) is the voxel-wise fMRI signal intensity. $\overset{-}{x}(l)=\int x_{r}(t,l)dt/T$ is the signal baseline value, which is the mean BOLD value crossing the time course in fMRI during an experimental resting condition and can be obtained through a temporal normalization procedure [19]. According to (5), the instantaneous power of fMRI signals can then be represented by

(8) $$\begin{array}{c} P_{\text{BOLD}}\left(t,l\right)=\frac{\partial E_{\text{BOLD}}\left(l\right)}{\partial t}={\left[x\left(t,l\right)-\overset{-}{x}\left(l\right)\right]}^{2}. \end{array}$$

Given that BOLD signals are composed of *M* independent components of brain activity according to the basic hypothesis underlying the ICA approaches used in fMRI studies, the instantaneous power of BOLD signals can be partitioned into *M* independent instantaneous powers of brain activity by sICA, as expressed by

(9) PBOLD(t,l)=at1Pactivation_1(1,l)+at2Pactivation_2(2,l)+⋯atmPactivation_m(m,l)+⋯atMPactivation_M(M,l).

Because the instantaneous power of fMRI signals can be considered as a linear mixture of each instantaneous power of brain activity and normally meets with the latent variables model of sICA, it is more suitable to use instantaneous power of fMRI signals partitioned by sICA than to use original fMRI signals.

## 3. Materials and Methods

### 3.1. Participants

Eighteen healthy volunteers (11 males; mean age 27.5 years; age range: 22–35 years) participated. The healthy subjects had no history of neurological or psychiatric disorders and were not on any medication for at least a month before the experiment. All the participants were right-handed as assessed using the Edinburgh handedness inventory [20]. The study was approved by the local Ethical Committee of Jinling Hospital, and written informed consent was obtained from all subjects prior to participating.

### 3.2. Experimental Paradigms

In the experiment, healthy subjects were scanned when performing a hand flexion task using their nondominant (left) hand [21]. The subjects were trained to grip the hand with a frequency of 1 Hz, and they practiced for 100 sec before the scan. A block design was used in the paradigm and the overall task consisted of 10 blocks, 5 task blocks alternating with 5 resting blocks, lasting for 200 sec with each block of 20 sec. During the functional scan, the subjects were instructed to grip the left hand when seeing a stationary cross presenting on the center of the screen throughout each task block and to remain still and fixate on a stationary asterisk throughout each resting block. The paradigm has been expounded in our precious study [21].

### 3.3. MRI Acquisition

Imaging data were acquired using a 1.5T GE MRI system (Signa) at Jingling Hospital, Nanjing, China. Foam padding was used to minimize head motion and improve participants comfort. fMRI time series of 100 repeated whole brain images were acquired in an orientation parallel to the AC-PC plane using a *T*_2_*-weighted GRE-EPI sequence. The sequence parameters were TR = 2000 ms, TE = 40 ms, FA = 80°, FOV = 24 × 24 cm^2^, 21 continuous slices with a thickness of 4 mm (no gap), and matrix size = 3.75 3.75 mm^2^. Anatomical images using a T1-Flair sequence (TR = 2019.3 ms, TE = 25.3 ms, interslice gap = 0.5 mm, and slice thickness = 4 mm) were acquired to facilitate the precise determination of the structures corresponding to the functional regions.

### 3.4. Preprocessing of Data

The fMRI data of each subject were first preprocessed using SPM8 software package (http://www.fil.ion.ucl.ac.uk/spm/), and spatial realignment was performed to remove head motion artifacts, and the functional scans were spatially normalized to a standard template (Montreal Neurological Institute) and resampled to 2 × 2 × 2 mm^3^. If the head motion and rotation parameters of a subject exceeded ±0.5 mm and ±0.5°, respectively, the data was excluded from further analysis. To increase the signal-to-noise ratio (SNR), the data were smoothed spatially using an isotropic Gaussian filter with a full width at half-maximum (FWHM) of 8 mm kernel.

### 3.5. ICA Analysis

Data from all participants were concatenated into a single dataset and reduced using two stages of principal component analysis (PCA) [7]. The optimal number of ICs was determined by a dimension estimation using the minimum description length (MDL) criterion [22]. ICA was then conducted to decompose the data from all subjects into different spatially independent components (ICs) with the FAST-ICA algorithm. For each IC, the time course corresponded to the waveform of a specific pattern of coherent brain activity, and the intensity of the pattern across the voxels is expressed in the associated spatial map. This analysis was repeated 50 times for assessing the repeatability of ICs [23]. To display the voxels that contributed most strongly to a particular IC, the intensity values in each spatial map were converted to *Z*-values (standard deviation of image) map [24]. The voxels with absolute ICA amplitudes larger than a specified amplitude threshold (i.e., |amplitude| > 2.5) were selected as the voxels with significant changes in brain activity [25].

### 3.6. Simulation

**Figure 1:**
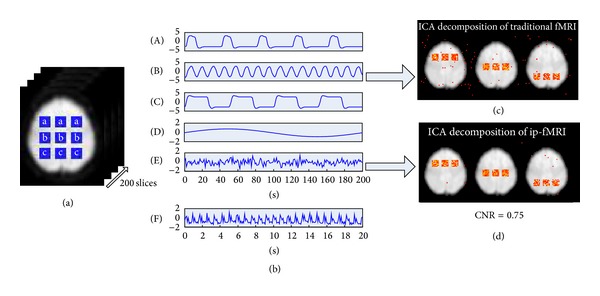
(a) AnEPI scan image with the selected nine regions of interest and simulated fMRI time courses. (b) Signals (A) and (B) (0.08 Hz) are the series of gamma variate functions simulating event-related and resting-state brain hemodynamics, respectively. Signal (C) is the convolution of an HRF and a square wave simulating a block-design fMRI signal. Signal (D) (0.005 Hz) is a sine wave simulating a slowly varying global baseline. Signal (E) is a Gaussian signal simulating the random white noise. Signal (F) (with a mean frequency of 1.2 Hz) is a cardiac signal simulating the structured noise. (c) Spatial components extracted by traditional fMRI. (d) Spatial components extracted by ip-fMRI. Voxels with amplitude values above threshold 2.5 are shown as the points in red to yellow color on the image.

A simulation was conducted to evaluate the performance of ICA decomposition of ip-fMRI. A slice of resting-state EPI scans (79 × 95 voxels) was replicated 200 times in order to simulate 200 time points of noise-free fMRI data. Nine 8 × 8 square blobs of voxels were selected for the simulation of localized activity changes (Figure 1(a)). The simulated time courses used in this section are shown in Figure 1(b). Three simulated signals (Signals (A)–(C)) were constructed to represent the brain hemodynamics for event-related activation (Signal (A)), resting-state activities (Signal (B)), and activation in block-designed paradigm (Signal (C)). A slowly varying baseline (Signal (D)) was added to all the voxels. To simulate the noisy environment in the brain, random noise and structured noise were mixed in the simulated data (all voxels in the brain area). The random noise (Signal (E)) follows a Gaussian distribution with a mean of 0 and a variance of 1. To simulate the structured noise, a cardiac signal (Signal (F)) which has an average frequency of 1.2 Hz was generated. The magnitudes of the signals and noises have been varied to adjust the contrast-to-noise ratio (CNR ≡ Δ*S*/*σ*_noise_). The CNRs ranged from 0.5 to 2, consistent with the CNR values reported in the fMRI literature [26].

The ip-fMRI and fMRI datasets were decomposed separately into spatially independent spatial patterns. Each pattern was associated with a temporal waveform, and only the components with the closest correlation between waveform and simulated true sources were considered. Examples of the spatial components decomposed by the two approaches are shown in Figures 1(c) and 1(d) with the CNR of 0.75. A receiver-operating characteristic (ROC) analysis was then conducted based on the spatial maps to determine the optimal component number, accuracy, and specificity of the two approaches.

## 4. Results

### 4.1. Simulation Results

**Figure 2:**
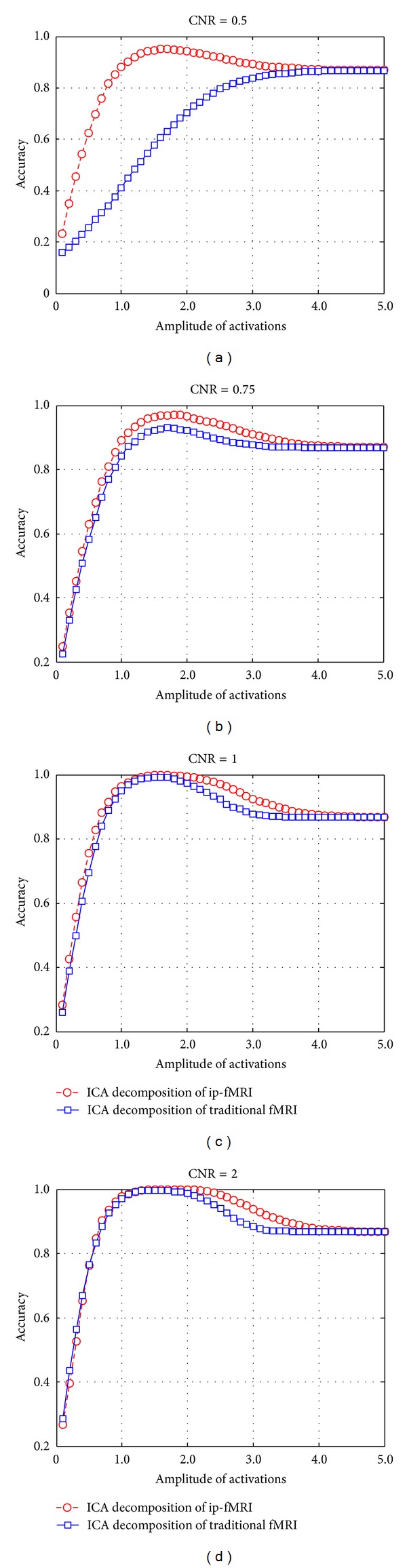
The accuracy analyses of ICA decomposition from ip-fMRI and traditional fMRI at CNRs of 0.5, 0.75, 1, and 2. The plots are the average accuracy curves of the fifty repeated procedures.

**Figure 3:**
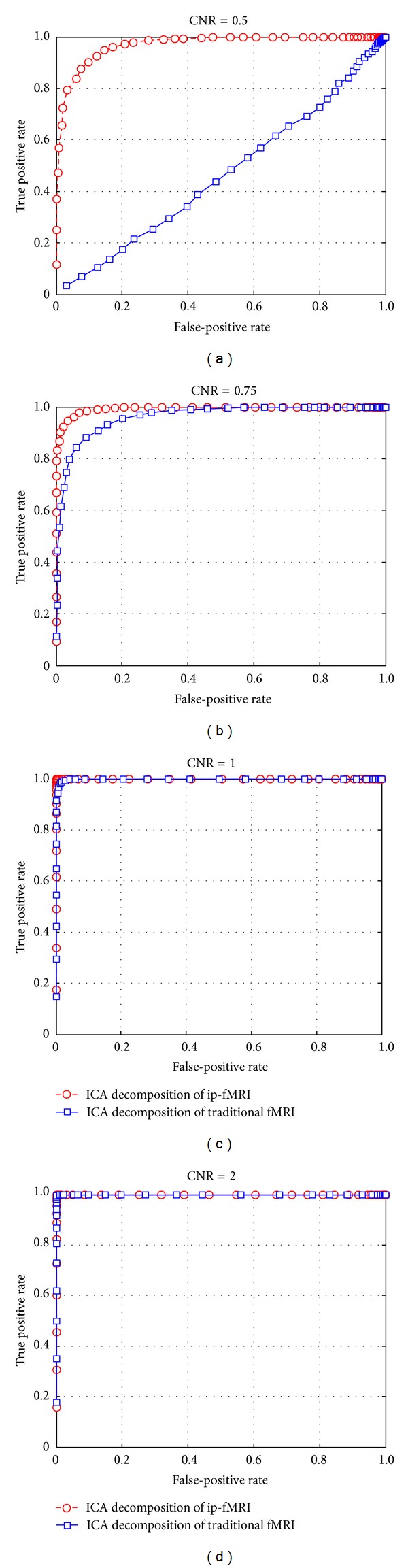
The ROC curves for ICA decomposition of ip-fMRI and traditional fMRI. Plotted are the mean ROC curves of the fifty repeated procedures. For the simulation with a CNR set to be 0.5, the area under the ROC curve (AUC) is 0.9751 and 0.4896 for ip-fMRI and traditional fMRI, respectively. Briefly, CNR = 0.75, AUC = 0.9932 (ip-fMRI) and 0.9723 (traditional fMRI); CNR = 1, AUC = 1 (ip-fMRI) and 0.9993 (traditional fMRI); CNR = 2, AUC = 1 (ip-fMRI) and 1 (traditional fMRI).

The accuracy was calculated for both the fMRI and ip-fMRI approaches based on our simulated dataset at different ICA amplitude levels and CNRs. Under almost all the conditions tested, ip-fMRI produced more suited results than the traditional fMRI, which was especially evident with low CNRs (Figure 2). In addition, each individual component separated from ip-fMRI was almost consistent when being used to localize the ROIs. Further comparisons of the ROC curves showed that those of ip-fMRI plotted were always over those of traditional fMRI (Figure 3). In terms of specificity, the AUC value of 1 represents a perfect test; while an AUC value of 0.5 or below just gives a chance discrimination. When CNR was set to be 0.5, the traditional fMRI failed to produce reliable results (AUC = 0.4896) while ip-fMRI still performed well (AUC = 0.9751). The simulation results indicate that the ICA decomposition from ip-fMRI outperforms that from the traditional fMRI, even though both of the approaches have an equal AUC value (i.e., = 1) when CNRs are set above 1.

### 4.2. Experimental Results

#### 4.2.1. Identification of the Functional Network Underlying Hand Movement

**Figure 4:**
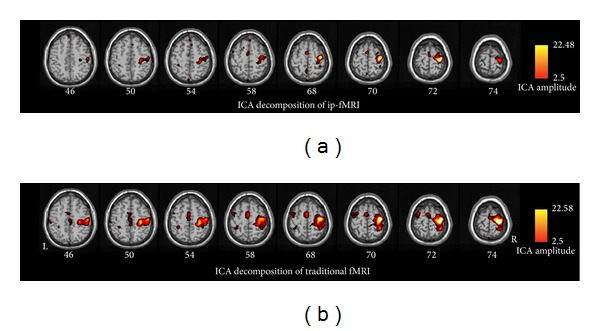
The motor functional networks of a random selected subject detected by ip-fMRI and traditional fMRI methods, respectively. (a) Motor networks detected by ip-fMRI. (b) Motor networks detected by traditional fMRI. The numbers beneath the axial MR image refer to Talairach coordinates.

The performance of the ICA decomposition of ip-fMRI was further evaluated for the detection of task-induced brain activation. A motor network underlying the left hand flexion task was identified using either approach, which consists of the contralateral primary motor (M1) and sensory (SI) cortices and the supplementary motor area [27]. However, the spatial component extracted from ip-fMRI from each subject became more anatomically focused or more specific to motor function than those from traditional fMRI. For the visualization, the motor component from a randomly selected subject was shown in Figure 4. Furthermore, the correlation coefficient between the temporal waveform associated with each spatial motor component and the designed ON-OFF paradigm was calculated. Two-sample *t*-test was employed to compare the results between ip-fMRI and traditional fMRI methods. Compared with the traditional fMRI, the result obtained from the two-sample *t*-test clearly showed significant difference (*t* = 3.0486, *P* = 0.0055, uncorrected). This enabled the identification of significant changes in the ip-fMRI method as compared with the traditional fMRI.

## 5. Discussion

ICA provides a method to separate signals “blindly” into spatially independent components, enabling exploratory analysis of fMRI data [2]. The key assumptions in sICA are that an fMRI dataset consists of a number of spatially independent components that are linearly mixed and spatially fixed. However, fMRI signals are actually nonlinear and are affected by many other artifacts such as those induced by head motion or physiological noises. Thus, fMRI signals may not automatically satisfy the commonly used latent ICA variables model.

To provide a better solution to this problem, we present an instantaneous power approach to resolving the ICA problem in fMRI analysis. We used instantaneous power to regularize fMRI signals such that the distribution of fMRI signal changes follows the temporal pattern in power distribution (defined in (8)). Then, the decompositions separated by ipICA can be simultaneously used to extract a variety of spatially independent components. The spatially independent but temporally coherent components represent the instantaneous power of each fMRI source signal (i.e., changes in brain activity). In other words, because the power of fMRI signals can be considered as a linear mixture of each instantaneous power, the components separated by ip-fMRI naturally satisfy the addition theorem to reflect different patterns of brain activity.

We have used a relatively realistic simulation based on a single volume of resting-state EPI data. This simulation has correct noise structures and spatial and temporal correlations with three artificial components added, which are shown in 9 dominant square blobs of the regions simulated. To make a visual comparison between the ICA results decomposed by ip-fMRI and traditional fMRI, Figures 1(c) and 1(d) show the components correlated to the simulated time series, respectively. For a given amplitude threshold, the ICA map from traditional fMRI tends to be noisier than that from ip-fMRI with the CNR of 0.75. Our results from the accuracy curves (Figure 2) and ROC curves (Figure 3) indicate that the performance of ip-fMRI is superior to that of the traditional fMRI under different CNRs or ICA amplitude values.

The proposed new ip-fMRI approach has been further validated using human data. A task-related fMRI experiment was provided for evaluating the new approach. When the actual mixtures are regularized through instantaneous power, the extracted spatial sources from the regularized results become more anatomically focused than those without the regulation (Figure 4) and their time courses become more fit into the designed paradigm for hypothesis testing.

In summary, we introduce a new ICA method based on the instantaneous power of fMRI signals to improve the decomposition and interpretation of fMRI data. The decomposed components by ip-fMRI represent the distribution of instantaneous power changes in fMRI signals. Combining the simulated and* in vivo *fMRI data, our results indicate that the spatially independent components decomposed from ip-fMRI are superior to those decomposed from the traditional fMRI in both accuracy and specificity for detecting the brain activity changes. We conclude that the ICA decomposition of ip-fMRI approach may provide a tool for the localization of energy changes in the brain, which may potentially be used to detect altered brain functions.
